# Integrated Analysis of microRNA-mRNA Expression in Mouse Lungs Infected With H7N9 Influenza Virus: A Direct Comparison of Host-Adapting PB2 Mutants

**DOI:** 10.3389/fmicb.2020.01762

**Published:** 2020-07-28

**Authors:** Yanna Guo, Nan Huang, Miao Tian, Menglu Fan, Qingzheng Liu, Zhiyuan Liu, Tongtong Sun, Jingjin Huang, Huizhi Xia, Yongzhen Zhao, Jihui Ping

**Affiliations:** MOE International Joint Collaborative Research Laboratory for Animal Health & Food Safety, Institute of Immunology, Nanjing Agricultural University, Nanjing, China

**Keywords:** influenza virus, miRNA, expression profile, miRNA microarray, transcriptome sequencing

## Abstract

MicroRNAs (miRNAs) are important regulators involved in the antiviral response to influenza virus infection, however, an analytical comparison of miRNA and mRNA expression changes induced by several H7N9 host-adapting PB2 mutants remains undone. Here, miRNA microarray and transcriptome sequencing of BALB/c mouse lungs infected with A/Anhui/1/2013 (H7N9) [hereafter referred to as H7N9/AH1-PB2-627K(WT)] and mutant variants with PB2 amino acid substitutions (avian-like H7N9/AH1-PB2-627E and mammalian-adapted H7N9/AH1-PB2-627E/701N) were directly compared. The results showed that influenza virus infection induced dysregulation of numerous host cell processes. In a miRNA-mRNA network associated with immunity, changes in the expression of 38 miRNAs and 58 mRNAs were detected following influenza virus infection. Notably, the miRNAs of mmu-miR-188-5p, mmu-miR-511-5p, mmu-miR-483-5p, and mmu-miR-690 were specifically associated with the replication of the avian-like virus H7N9/AH1-PB2-627E. Likewise, the miRNAs of mmu-miR-691, mmu-miR-329-3p, and mmu-miR-144-3p were specifically associated with the mammalian-adapted virus H7N9/AH1-PB2-627E/701N. Finally, the miRNAs of mmu-miR-98-5p, mmu-miR-103-3p, mmu-miR-199a-5p, and mmu-miR-378a-3p were specifically associated with H7N9/AH1-PB2-627K(WT) virus replication. This is the first report of comparative integration analysis of miRNA-mRNA expression of these three H7N9 influenza viruses with different host-adapting PB2 mutations. Our results highlight potential miRNAs of importance in influenza virus pathogenesis.

## Introduction

Avian influenza viruses (AIVs) are an important zoonotic pathogen that can pose a serious threat to the development of the poultry industry and human health due to their rapid mutation rate and their ability to adapt to human hosts. There have been large-scale outbreaks of influenza virus infection in humans during the past few decades, especially the four influenza pandemics. Adaptive mutations allow AIVs to efficiently replicate within new hosts and to achieve host-host transmission. For influenza viruses, the hemagglutinin receptor protein is one of the major factors determining host range. AIVs that transition hemagglutinin receptor preference from avian-type (α-2,3-linked Sialic acid) to human-type (α-2,6-linked Sialic acid) are vital to human adaptation (Manz et al., 2013; Zaraket et al., 2013) and sustained transmission among humans (Herfst et al., 2012; Imai et al., 2012). Another prominent contributor to influenza virus tropism is the viral polymerase complex. Many key adaptive mutations in viral polymerase proteins are known to significantly enhance polymerase activity in mammalian hosts and otherwise improve mammalian cell permissively (Naffakh et al., 2008; Ping et al., 2011). It is well known that the adaptive mutations E627K and D701N in the influenza virus PB2 protein are two important markers for the cross-species transmission of AIVs to mammals or humans (Hatta et al., 2001; Li et al., 2005; Kong et al., 2019). The PB2-E627K mutation enhances its pathogenicity in mice by enhancing the binding capacity of importin α7 to influenza vRNPs (Resa-Infante et al., 2014). Recent studies indicate that species-specific differences in host ANP32A proteins were key factors limiting the efficiency of AIV infection of mammalian cells. Avian ANP32A can increase AIV polymerase activity, whereas mammalian ANP32A does not (Long et al., 2016). Furthermore, in AIVs, the PB2-D701N mutation can partially compensate for the lack of PB2-E627K and enable the transfer of vRNPs from importin-α3 to importin-α7. This adaptation results in enhanced replication and pathogenicity of AIVs in mammals (Gabriel et al., 2008, 2011; Steel et al., 2009).

MicroRNAs (miRNAs) are a class of 19∼25 nt non-coding regulatory molecules that function primarily by causing target mRNA degradation or translational inhibition at the post-transcriptional level with complete or incomplete complementary binding to the 3′UTR region of the target gene (Bartel, 2004, 2018). As a novel regulatory molecule, miRNAs are involved in many biological processes, including growth and development, differentiation, apoptosis, cancer, and more (Ambros, 2004; Lu et al., 2005). It has also been reported that many host miRNAs are crucial to antiviral immune responses (Lecellier et al., 2005; Huang et al., 2007; Pedersen et al., 2007; Jennifer and Bryan, 2009). Each miRNA molecule can act on multiple host target transcripts, and each target mRNA can also be regulated by multiple miRNAs. So, it follows that ensuring the normal functioning of the miRNA-mRNA regulatory network is important for maintaining the homeostasis of the cellular immune system. Dysregulation of this network may lead to autoimmune disorders or susceptibility to disease (Baltimore et al., 2008; Chen et al., 2013, 2016; Danger et al., 2014).

In recent years, increasing evidence confirms that miRNAs play a crucial role in promoting or inhibiting the replication of influenza virus (Ma et al., 2016). miRNAs can have different effects on influenza virus replication due to their targeted action on different transcripts. For example, miR-146 can inhibit the expression of *IRAK1*, *MAPK3*, and tumor necrosis factor receptor-associated factor 6 (*TRAF6*); this downregulation promotes the activation of NF-κB, thereby inhibiting viral replication (Buggele et al., 2012; Terrier et al., 2013). As an attenuator of cellular antiviral immunity, hsa-miR-340-5p is known to target the mRNAs of the antiviral proteins RIG-I and OAS2; this miRNA was shown to be downregulated in influenza A virus-infected A549 cells resulting in significantly inhibited influenza virus replication (Zhao et al., 2019). miR-3145 is able to target the viral PB1 gene and cause its degradation to inhibit the replication of influenza virus (Khongnomnan et al., 2015). miR-302c can inhibit the expression of IFN-β by inhibiting the transfer of NF-κB from the cytoplasm into the nucleus, which is beneficial to the replication of influenza virus (Gui et al., 2015). The expression of miR-29c is up-regulated after infection with H1N1 and H3N2 viruses. This miRNA can protect the expression of A20 mRNA, which correlates with inhibition of both NF-κB activity and the production of cytokines, leading to enhanced influenza virus replication (Zhang et al., 2014). Studies show that miR-485 has dual functions for influenza virus replication. When cells are infected with a low dose of H5N1 influenza virus, the host miR-485 can target RIG-I mRNA to cause its degradation, resulting in inhibition of the antiviral immune response and promoting viral replication. In contrast, when infected with high doses of H5N1 influenza virus, cellular miR-485 can directly target the influenza virus PB1 gene, thereby inhibiting the formation of the viral polymerase which ultimately leads to inhibited viral replication (Ingle et al., 2015). Furthermore, host miRNAs also act a pivotal part in regulating the pathogenicity of influenza viruses. Different pathogenic influenza viruses can induce different miRNA expression profiles (Li et al., 2010, 2011; Rogers et al., 2012), and different host genetic backgrounds also have a certain impact on miRNA expression during influenza virus infection (Wu et al., 2013; Bao et al., 2018). Although the miRNA expression of C57BL/6J and DBA/2J mice infected with H7N9 influenza virus has been studied, a comprehensive analysis of host miRNA and mRNA expression profiles in the lungs of mice infected with mammalian H7N9/AH1-PB2-627K(WT) influenza virus and PB2 host-adapted mutants has not been reported.

In this study, we used miRNA microarray and transcriptome sequencing technology to study differential expression of miRNA and mRNA in lung tissues infected with H7N9/AH1-PB2-627K(WT) influenza virus and PB2 mutants with key amino acid differences at positions 627 or 701. The difference in virulence of influenza strains leads to differences in pathogenicity; how these known PB2 mutations specifically affect the cellular defenses in a mouse lung model remains unknown. This research will help deepen our understanding of the important role of miRNAs in influenza virus pathogenesis, provide data to support the screening for key host miRNAs involved in the regulation of influenza virus replication, and lay a foundation for elucidating the mechanism of AIV cross-host infection in mammals at the non-coding RNA level.

## Materials and Methods

### Virus

The eight gene segments of A/Anhui/1/2013 (H7N9/AH1-PB2-627K(WT) virus used in this study were synthesized by Wuhan Jinkairui Company and then cloned into the transcription vector pHH21. PB2 gene mutant plasmids H7N9/AH1-PB2-627E and H7N9/AH1-PB2-627E/701N were obtained by site-directed mutagenesis. H7N9/AH1-PB2-627K(WT) and its mutant viruses applied in this research were rescued through reverse genetics system (Neumann et al., 2005). Briefly, 1 μg of each of the four protein-expressing plasmids and 0.2 μg of each of the vRNA expressing plasmids (pHH21-AH1-PB1, pHH21-AH1-PA, pHH21-AH1-HA, pHH21-AH1-NP, pHH21-AH1-NA, pHH21-AH1-M, pHH21-AH1-NS, and pHH21-AH1-PB2 627K or the PB2 mutant plasmids (pHH21–AH1-PB2 627E or pHH21–AH1-PB2 627E/701N) were combined with 12 μl TransIT-293 transfection reagent (Mirus) (2 μl per μg DNA), and the mixture was incubated at room temperature for 30 min and then transferred to a 60–70% confluent monolayer of 293T cells on a 35 mm dish (Thermo Fisher Scientific). At 48 h post-transfection, the 293T supernatants were inoculated into 10-day-old SPF embryonated chicken eggs for virus propagation.

The proliferated viruses were genetically identified without mutation via Sanger sequencing and then stored at −80°C and virus titer was detected via the 50% egg-infective dose (EID_50_) method. Nanjing agricultural university approved work with these viruses in negative pressure enhanced BSL-2 + plus containment.

### Mice Infection Experiments

Specific pathogen-free 4- to 6-week-old female BALB/c mice were supplied by Xipuer-bikai Laboratory Animal Ltd., Co. (Shanghai, China). All mice were divided into four groups randomly with four mice in each group and kept in individually ventilated cage (IVC), the system supplies HEPA-filtered air to each and every rodent micro-barrier cage. Exhaust air is drawn directly from the cage and HEPA filtered prior to being returned to the room. The mice were anesthetized with isoflurane and intranasally inoculated with 5 × 10^5^ EID_50_ (in 50 ul) of H7N9/AH1-PB2-627K(WT) and its mutant virus. Body weight and mortality was monitored for 14 days. All animal work was carried out in a enhanced BSL2 + laboratory. The researchers who working in BSL2 + lab wore Tyvek and powered purified air respirators.

For pathological examination, BALB/c mice were inoculated with 5 × 10^5^ EID_50_ influenza A virus, and lungs were collected on days 3 and 5 post-infection, the lung tissues were collected and fixed in 10% neutral-buffered formalin overnight and then made paraffin sections by a microtome device (Leica, RM2245, Germany). Through deparaffinization and hydration, slides were stained with hematoxylin and for 4 min, then washed by water for 5 min, followed handled by different gradient alcohol and then stained in eosin for 15 s. Following dehydration through alcohol and xylene, the section was sealed with neutral gum. Then the H&E stained slides were observed a under light microscope (DP73, Olympus, Tokyo, Japan) for pathological changes.

### Virus Titrations

The viruses used for mice infection experiment were titrated by the EID_50_. The virus samples were successively diluted 10-fold, and then the different dilutions were inoculated into 10-day-old SPF embryonated chicken eggs, 100 μl/egg. The allantoic fluid was collected 48 h post-inoculation at 37°C and virus propagation was confirmed by hemagglutination assays. The EID_50_ value was calculated using the Reed-Muench method based on HA value of each egg. The viral titers of infected mouse lung tissue were determined by 50% tissue culture infective dose (TCID_50_) in MDCK cells. Briefly, the mouse lungs were homogenized within 1 ml PBS, and the supernatants were serially diluted 10 times before adding to 95% confluent MDCK cells in 96 well plates. After incubation at 37°C for 1 h, the virus solution was discarded, the plates were washed twice with sterilized PBS, and then serum-free DMEM maintenance medium containing 1 ug/ml N-tosyl-L-phenylalanine chloromethyl ketone (TPCK) treated trypsin was added. The cells were fixed with 4% formaldehyde solution at 48 h post-infection and then successively incubated and washed with primary and fluorescently-labeled secondary antibodies. The number of fluorescence positive wells were counted and virus titers were calculated using Reed-Muench method. Three independent experiments in each group were performed.

### Immunofluorescence Staining

Immunofluorescence staining was performed as previously described (Ping et al., 2010). Briefly, BALB/c mice were inoculated with 5 × 10^5^ EID_50_ influenza A virus, and lungs were collected on days 3 and 5 post-infection and fixed and embedded in paraffin for sectioning at a thickness of 4 μm. Virus antigen was detected by incubating with primary anti-H7N9 HA antibody (1:100, Sino Biological) in buffer (10 mM PBS containing 3% bovine serum albumin and 0.3% Triton X-100). After being washed three times with 10 mM PBS, the slides were incubated with A647 goat anti-rabbit secondary antibody (1:100, Invitrogen). The slides were then washed, and nuclei were stained by incubation with 4′,6-diamidino-2-phenylindole (DAPI) for 10 min. Images were taken by a BX53 Olympus DP73 fluorescence microscope.

### RNA Isolation

Mice lung tissues infected by H7N9/AH1-PB2-627K(WT) and its mutant viruses were collected 3 days post-infection and were homogenized using a tissue homogenizer (Retsch, Germany) to collect tissue supernatant subsequently. Lysis/binding buffer was added into the supernatant (1 ml Lysis/binding buffer per 0.1 g tissue) to lysate cells. The total RNA was extracted using an mirVana^TM^ RNA isolation kit (Ambion). The concentration of RNA was measured by a NanoDrop 2000 Spectrophotometer (Thermo). And the RNA integrity were then assessed via Agilent Bioanalyzer 2100 (Agilent Technologies).

### miRNA Microarray and miRNA Data Analyses

Total RNA samples from H7N9/AH1-PB2-627K(WT) virus-infected mouse lungs were first dephosphorylated and denatured, then labeled with Cyanine-3-CTP by ligation following the manufacturer’s instructions of miRNA complete labeling and Hyb Kit (5190-0456, Agilent). Next, these labeled miRNAs were purified. The purified miRNAs were then hybridized to the microarray which was washed according to the protocol of the Gene Expression Wash Pack (5188-5327, Agilent). Finally, the microarray was scanned by Agilent Scanner G2505C (Agilent Technologies). The Agilent Mouse miRNA microarray, Release 21.0 (8 × 60K, Design ID:070155) contains 1881 probes for mouse mature miRNAs was applied by OE Biotech Co., Ltd. (Shanghai, China). The miRNA array scan images were obtained by the Feature Extraction software (version 10.7.1.1, Agilent Technologies), the generated raw data were standardized by GeneSpring software (version 14.8, Agilent Technologies) for subsequent data analysis. The probes that had at least 100.0 percent of samples in any 1 condition out of 2 conditions with flags in “Detected” were chosen for further data analysis. Differentially expressed miRNAs were then identified through fold change as well as had their *P* value calculated using student’s *t*-test. The threshold set for up- and down-regulated genes was a fold change ≥2.0 and a *P* value ≤ 0.05. Target genes of differentially expressed miRNAs were defined by the intersection predicted using three databases (TargetScan, microRNA.org, and PITA). GO analysis and KEGG analysis were applied to determine the roles of these target genes. Hierarchical Clustering was performed to show the distinguishable miRNAs expression pattern among samples.

### Transcriptome Sequencing Process

Total RNA from lung tissues of infected mice was extracted and then quantified by NanoDrop ND-2000 Spectrophotometer. Ribosomal RNA was digested by using TruSeq Stranded Total RNA with Ribo-Zero Gold kit, then digested RNA into short fragments; cDNA was synthesized by reverse transcription method using interrupted RNA as template. Subsequently, purified cDNA was end-repaired, A-tailed, and ligated to the sequencing linker, PCR amplification followed. After the RNA libraries were constructed, they were qualified by Agilent 2100 Bioanalyzer and then Illumina sequencing was performed. The original image data obtained by high-throughput sequencing were converted into original sequencing sequences (Raw Reads) by base call analysis. Then quality assessment of raw reads was carried out using Trimmomatic software, which yielded clean reads. Sequence alignment of clean reads with a specified reference genome using HISAT2 obtained positional information on a reference genome. The expression of genes was calculated using the FPKM method (Fragments Per kb Per Million Reads). Differentially expressed mRNAs were selected with the screening criteria *P* value of <0.05 and fold change of >2 or <0.5. Cellular component (CC), Biological process (BP), Molecular function (MF) enrichment analysis of differentially expressed genes between samples was carried out using the fisher algorithm. Pathway analysis of differentially expressed genes was applied using KEGG database.

### qPCR Verification of Differentially Expressed miRNA

The expression of the selected miRNAs was detected by the stem-loop method. Mature miRNAs were reverse-transcribed in each group with the corresponding HiScript II 1st Strand cDNA Synthesis Kit reverse transcription kit. U6 was used as an internal reference. The qPCR assay was performed at 95°C for 5 min, then 95°C 10s, and 60°C 30s for 40 cycles, followed by a melting curve to record the specific PCR product. The corresponding primers were shown in Supplementary Table S1.

### Statistical Analysis

In the qPCR experiment, the relative expressions were calculated by 2^–ΔΔCT^ method with GAPDH as an endogenous control gene for mRNA and U6 as a internal control for miRNA. Each reaction was conducted in triplicate.

## Results

### Pathological Analysis of Lung Tissues in Mice Infected With H7N9/AH1-PB2-627K(WT) and Its Mutant Influenza Viruses

**FIGURE 1:**
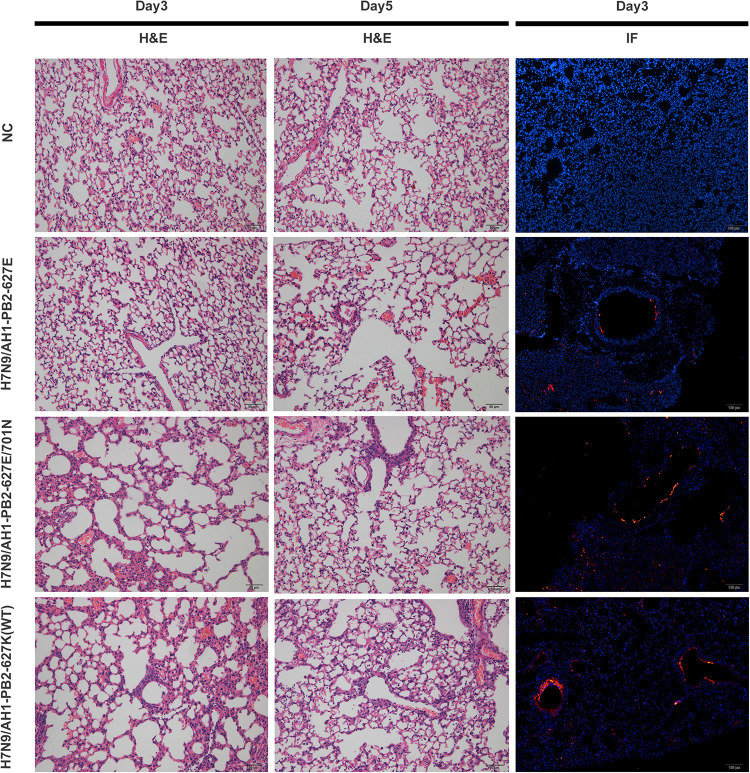
Pathological analysis of lung tissues of mice infected with H7N9/AH1-PB2-627K(WT) and its mutant viruses on day 3 or 5 post-infection. Scale bars: HE (50 μm); IF (100 μm).

To investigate the pathogenicity of H7N9/AH1-PB2-627K(WT) and PB2 mutants in a mouse model, 6-week-old female BALB/c mice were infected with an intranasal viral dose of 5 × 10^5^ EID_50_, the lung tissues of three infected mice from each group were collected on the 3rd and 5th day post-infection, and including a PBS infection group as negative control (hereafter designated as NC group). The histopathological lesions and damaging extent of lung tissue infection was observed by Hematoxylin and eosin (H&E) staining and immunofluorescent staining. The H&E results showed lung pathological lesions were mildest for the H7N9/AH1-PB2-627E infection group, which was similar to the NC group. Infection from H7N9/AH1-PB2-627K(WT) and H7N9/AH1-PB2-627E/701N viruses caused increased lung lesions with a large area of alveolar wall thickening, interstitial adhesion, capillary congestion, and a noticeable increase in inflammatory cell infiltration in the alveolar region. The clinical symptoms were much milder on the 5th day post-infection when compared to the 3rd day. Immunofluorescent staining showed that there were limited viral antigens detected in the bronchi for H7N9/AH1-PB2-627E at day 3; by comparison, at day 3 much more antigen staining was detected in the lungs infected with the other two PB2 mutant viruses. The results were highly consistent with the H&E results (Figure 1). The PB2 genotypes of the three viruses are summarized in Table 1.

**TABLE 1:** Amino acids at PB2 position 627 and 701 of viruses used in this study.

	**Residue 627 of PB2**	**Residue 701 of PB2**
H7N9/AH1-PB2-627K(WT)	K	D
H7N9/AH1-PB2-627E/701N	E	N
H7N9/AH1-PB2-627E	E	D

### Pathogenicity Analysis of H7N9/AH1-PB2-627K(WT) and Its Mutant Virus in Mice

**FIGURE 2:**
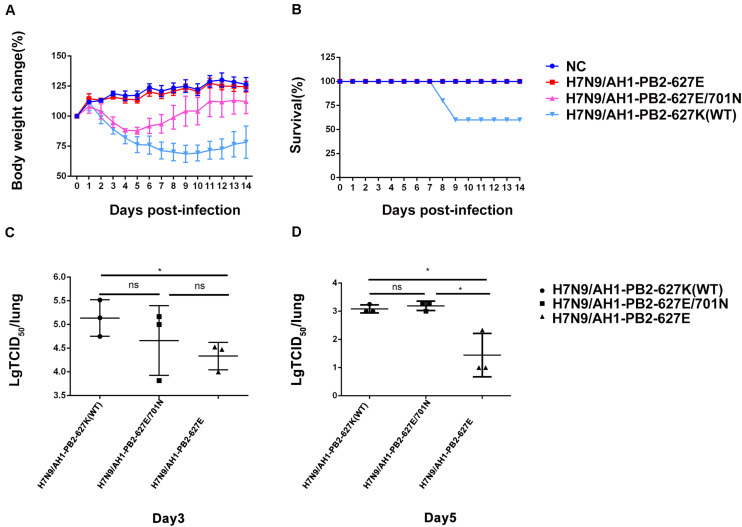
Pathogenicity analysis of mice infected with H7N9/AH1-PB2-627K(WT) and its mutant viruses. Four mice per group were inoculated intranasally with the indicated doses of three virus and monitored for survival rate **(A)** and body weight changes **(B)** for 14 days. The viral titers of homogenized lung tissue on day 3 post-infection **(C)** and day 5 post-infection **(D)** of all three infected groups were measured by TCID50. The results shown are means ± standard deviations (SD) (*n* = 3) (error bars) from three individual mice.

To further compare the virulence of the three PB2 variants in mice, *in vivo* infection experiments were performed with the same infection dose as above, and the mouse body weight change and mortality were monitored for 2 weeks post-infection (Figure 2). It was observed that the body weight loss of mice infected with H7N9/AH1-PB2-627K(WT) virus achieved ∼30% on day 9 post-infection and then recovered slightly, while the mortality rate reached 40% on the same day. For the group infected with H7N9/AH1-PB2-627E/701N virus, mouse body weight loss reached a maximum average loss of 12% on the 5th day, but no mortality was observed; no body weight loss or mortality were observed following infection with H7N9/AH1-PB2-627E virus (Figures 2A,B). The data revealed that both the PB2-627K and 701N mutations could significantly increase the virulence of H7N9/AH1-PB2-627K(WT) virus in mice. Additionally, the viral yield of the three viruses in mice lungs were also assessed by means of TCID_50_ in MDCK cells on 3rd and 5th day post-infection. The results showed that the viral titers in the lungs of all three infected groups were higher on day 3 than day 5, and the viral shedding of H7N9/AH1-PB2-627E in mice lungs was 2.5 times lower than either the PB2-627K or 701N mutant viruses at day 5 (Figures 2C,D). Collectively, these mice infection data demonstrated that the virulence of H7N9/AH1-PB2-627K(WT) and H7N9/AH1-PB2-627E/701N were higher than H7N9/AH1-PB2-627E in mice.

### Analysis of miRNA Expression Profiles in Lung Tissues of Mice Infected With H7N9/AH1-PB2-627K(WT) and Its PB2 Variants

**FIGURE 3:**
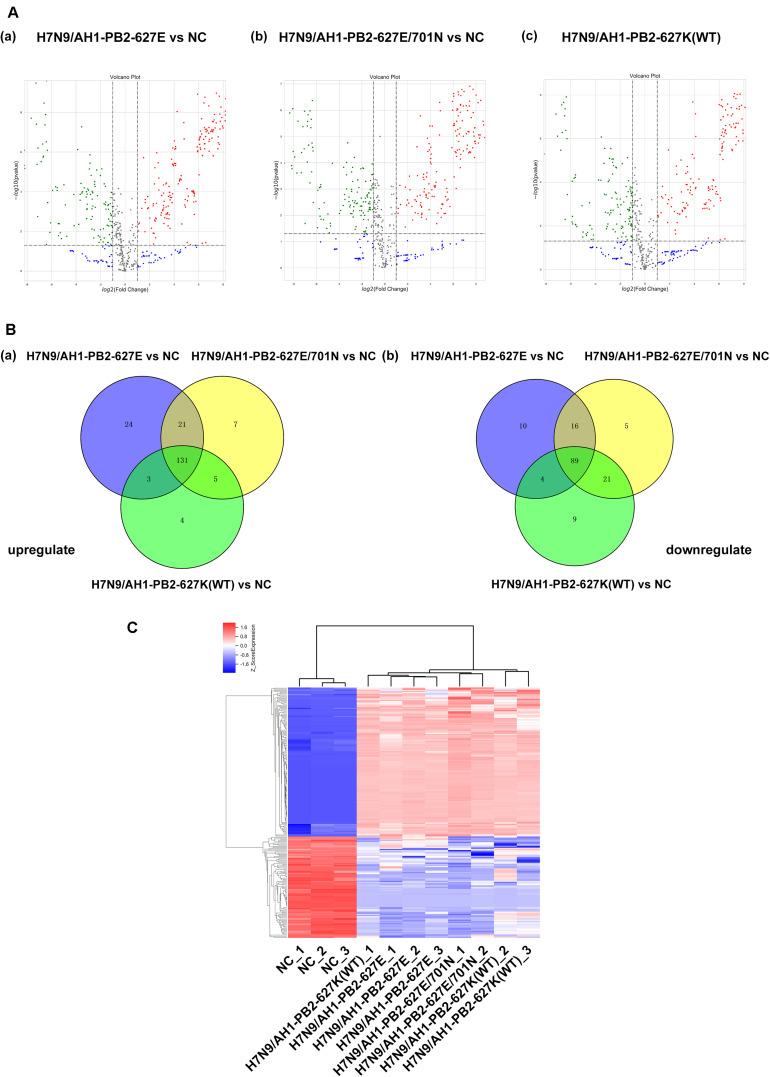
Global analysis of microRNA expression profiles in mice lung infected with H7N9/AH1-PB2-627K(WT) and its mutant influenza viruses compared with negative control group (NC). **(A)** The scatter diagram shows the distribution of differential expression of miRNAs in different influenza virus infected groups relative to the NC group. Red and green dots denote up- and down-regulated differentially expressed miRNAs, respectively. **(B)** The Venn diagram shows the number of the common and specific differentially expressed miRNAs in the three influenza virus infected groups: H7N9/AH1-PB2-627E vs. NC (blue circle), H7N9/AH1-PB2-627E/701N vs. NC (yellow circle), and H7N9/AH1-PB2-627K(WT) vs. NC (green circle). **(C)** Hierarchical clustering presents the common differentially expressed miRNAs compared with NC group. Up-regulated miRNAs are shown in red and down-regulated miRNAs are shown in blue.

Literature has already confirmed that miRNAs play a transformative role in promoting or inhibiting the replication of influenza virus *in vivo* and *in vitro*. Gene chip technology was employed to identify miRNA expression in mice infected with influenza viruses having the previously mentioned key substitutions at PB2-627 and PB2-701 (≥2-fold change and *p* ≤ 0.05 as screening criteria). The results showed that the virus-infected groups H7N9/AH1-PB2-627E, H7N9/AH1-PB2-627E/701N, and H7N9/AH1-PB2-627K(WT) detected 298, 295, and 266 differentially expressed miRNAs, respectively, compared to NC (Figure 3A). In the H7N9/AH1-PB2-627E mutant virus-infected group, 179 (60%) up-regulated miRNAs and 119 (40%) down-regulated miRNAs were significantly expressed; 164 (56%) up-regulated miRNAs and 131 (44%) down-regulated miRNAs were detected in H7N9/AH1-PB2-627E/701N mutant virus-infected group; and 143 (54%) up-regulated miRNAs and 123 (46%) down-regulated miRNAs were detected in the H7N9/AH1-PB2-627K(WT) virus-infected group. More upregulated miRNAs were induced than downregulated miRNAs in the three virus-infected groups, suggesting that miRNAs expressed in infected lung tissue were mainly upregulated during influenza virus infection. Some miRNAs shared similar patterns of up-regulation and down-regulation irrespective of which virus-infected group was analyzed; these were called common differentially expressed miRNAs. There were also some differentially expressed miRNAs that were induced only during infection with specific PB2 variants; these were called strain-specific differentially expressed miRNAs. On day 3 post-infection for all three infected groups, H7N9/AH1-PB2-627E, H7N9/AH1-PB2-627E/701N, and H7N9/AH1-PB2-627K(WT), a total of 220 common differentially expressed miRNAs were significantly changed compared to NC, of which 131 were up-regulated and 89 were down-regulated. The detailed information of common differentially expressed miRNAs can be seen in Supplementary Tables S2, S3. Venn diagrams showed that there were 34, 12, and 13 strain-specific differentially expressed miRNAs observed in the three different virus-infected groups (Figure 3B, and Tables 2, 3). Furthermore, hierarchical clustering analysis was used to show the expression profile of 220 common differentially expressed miRNAs in different treatment groups. The results indicated that these miRNAs were divided into two major clusters, one uninfected group containing all the negative controls, and one containing all three infected groups. The data illustrated that the expression patterns of these common miRNAs were significantly distinct from NC group, and three infected groups were similar but disparate with each other (Figure 3C). Validation of expression levels for randomly selected miRNAs was demonstrated using qPCR (Supplementary Figure S1); the qPCR results were consistent with the expression trends seen in the transcriptome sequencing results for the selected miRNAs. Besides, several common and specific miRNAs screened out also be performed to verify the impact of these miRNAs on viral replication, qPCR results showed that some miRNAs could promote the expression of viral NP genes (Supplementary Figure S2).

**TABLE 2:** Specific upregulated miRNAs in lung tissues of mice induced by H7N9/AH1-PB2-627K(WT) and its mutant viruses.

**Category**	**Specific miRNAs**
H7N9/AH1-PB2- 627E vs. NC	mmu-miR-1231-3p, mmu-miR-15a-3p, mmu-miR-3057-3p, mmu-miR-33-3p, mmu-miR-425-3p, mmu-miR-449a-5p, mmu-miR-511-5p, mmu-miR-547-5p, mmu-miR-5617-3p, mmu-miR-6351, mmu-miR-6909-3p, mmu-miR-6958-3p, mmu-miR-6959-3p, mmu-miR-6981-3p, mmu-miR-6990-5p, mmu-miR-6996-5p, mmu-miR-7012-3p, mmu-miR-7032-3p, mmu-miR-7074-5p, mmu-miR-7221-5p, mmu-miR-7674-3p, mmu-miR-7a-2-3p, mmu-miR-92b-5p, mmu-miR-9769-3p
H7N9/AH1-PB2- 627E/701N vs. NC	mmu-let-7b-3p, mmu-miR-144-3p, mmu-miR-329-3p, mmu-miR-3547-3p, mmu-miR-669m-3p, mmu-miR-675-3p, mmu-miR-7034-3p
H7N9/AH1-PB2- 627K(WT) vs. NC	mmu-miR-135a-1-3p, mmu-miR-203-3p, mmu-miR-3960, mmu-miR-714

**TABLE 3:** Specific downregulated miRNAs in lung tissues of mice induced by H7N9/AH1-PB2-627K(WT) and its mutant viruses.

**Category**	**Specific miRNAs**
H7N9/AH1-PB2- 627E vs. NC	mmu-miR-188-5p, mmu-miR-1897-5p, mmu-miR-1982-5p, mmu-miR-483-5p, mmu-miR-6385, mmu-miR-6538, mmu-miR-690, mmu-miR-7047-5p, mmu-miR-7118-5p, mmu-miR-7235-5p
H7N9/AH1-PB2- 627E/701N vs. NC	mmu-miR-1927, mmu-miR-6378, mmu-miR-691, mmu-miR-7218-5p, mmu-miR-7227-3p
H7N9/AH1-PB2- 627K(WT) vs. NC	mmu-miR-103-3p, mmu-miR-199a-5p, mmu-miR-200b-3p, mmu-miR-30a-3p, mmu-miR-378a-3p, mmu-miR-378b, mmu-miR-429-3p, mmu-miR-449a-5p, mmu-miR-98-5p

### Analysis of mRNA Expression Profiles in Lung Tissues of Mice Infected With H7N9/AH1-PB2-627K(WT) and Its Mutant Viruses

**FIGURE 4:**
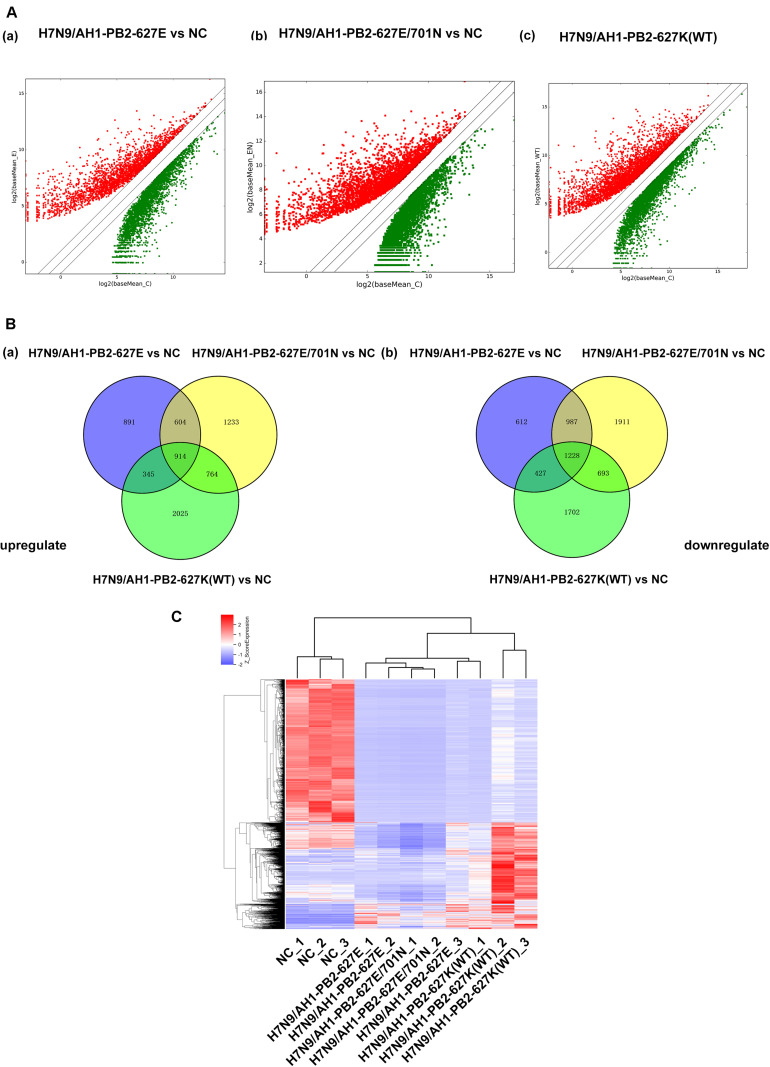
Global analysis of mRNA expression profiles in mouse lung infected with H7N9/AH1-PB2-627K(WT) and its mutant influenza viruses compared with negative control group (NC). **(A)** The scatter diagram showing the distribution of differential expression of mRNAs in different influenza virus infected groups relative to the NC group. Red and green dots represent up- and down-regulated differentially expressed mRNAs, respectively. **(B)** The Venn diagram shows the number of common and specific differentially expressed mRNAs in the three influenza virus infected groups: H7N9/AH1-PB2-627E vs. NC (blue circle), H7N9/AH1-PB2-627E/701N vs. NC (yellow circle), H7N9/AH1-PB2-627K(WT) vs. NC (green circle). **(C)** Hierarchical clustering depicts the common differentially expressed mRNAs compared with negative control group.

Transcriptome sequencing technology was used to analyze the mRNA expression profiles of mouse lung tissues infected with the three PB2-variant viruses (≥2-fold change and *p* ≤ 0.05 as screening criteria). For the three virus-infected groups, differentially expressed mRNAs matching these criteria were depicted in volcano maps (Figure 4A). The results showed a total of 6008 differentially expressed genes were significantly changed in H7N9/AH1-PB2-627E mutant virus infected mice compared to the NC group, which was significantly higher than the number of differentially expressed miRNAs. Of these differentially expressed mRNAs, 2754 (46%) were up-regulated and 3254 (54%) were down-regulated. There were in total of 8274 differentially expressed mRNAs were identified in H7N9/AH1-PB2-627E/701N mutant virus-infected group. Of these, 3455 (42%) were up-regulated mRNAs and 4819 (58%) were down-regulated mRNAs. Finally, there were 8083 differentially expressed mRNAs in the H7N9/AH1-PB2-627K(WT) virus-infected group, of which 3988 (50%) were up-regulated mRNA, and 4050 (50%) were down-regulated mRNA. Venn diagrams show that 2142 common differentially expressed mRNAs were significantly changed in the lung tissues of mice infected with any of the three different viruses. Of these, 914 mRNAs were up-regulated and 1228 mRNAs were down-regulated, the detailed information about these common mRNAs were shown in Supplementary Tables S4, S5. After infection of the H7N9/AH1-PB2-627E, H7N9/AH1-PB2-627E/701N and H7N9/AH1-PB2-627K(WT) virus, 1503, 3144, and 3712 specific differentially expressed mRNAs were identified by the screening criteria within each group, respectively (Figure 4B). It was observed that the higher the virulence in mice, the more differentially expressed mRNAs were identified. In addition, cluster analysis of these 2142 differentially expressed mRNAs indicated that the expression of differentially expressed mRNAs were significantly different between the virus-infected groups and the NC group (Figure 4C). Validation of these trends was performed on randomly selected genes’ mRNA expression patterns, using qPCR (Supplementary Figure S3).

### Functional Enrichment Analysis of Common Differentially Expressed miRNAs

**FIGURE 5:**
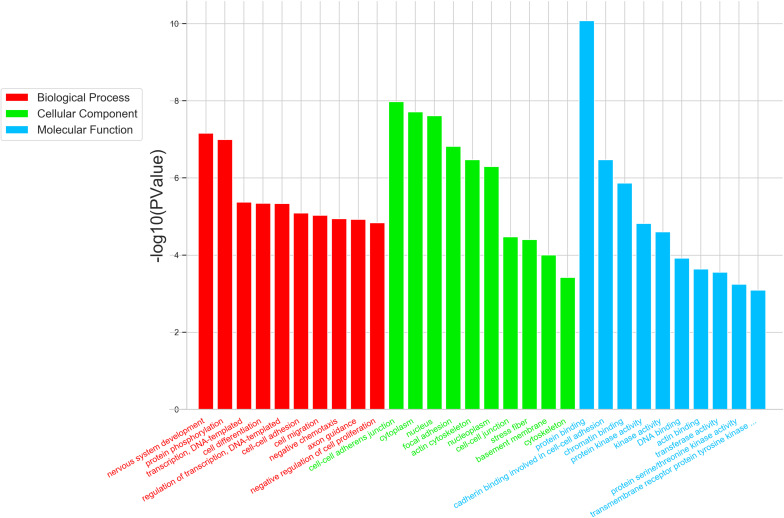
Top 10 significantly enriched GO terms of the overlapping target genes of common differentially expressed miRNAs were depicted in the three virus infected groups. GO analysis showed that the intersectional putative target genes of common miRNAs were involved in three parts of GO terms: Biological Process (Red), Cellular Component (Green), and Molecular Function (Blue). The expression trend of putative target genes involved in this GO term is inversely related to the expression of their targeting miRNAs.

**FIGURE 6:**
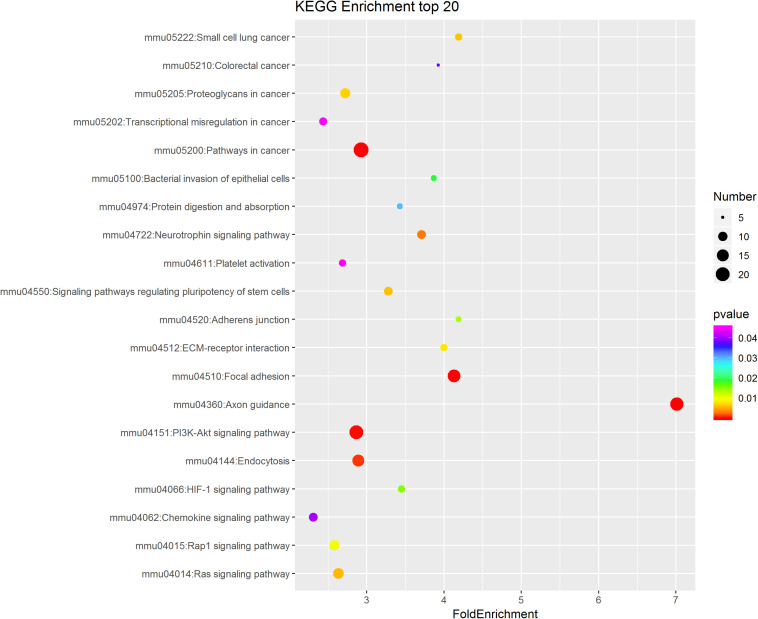
KEGG pathway analysis of the intersectional putative target genes of common miRNAs in the three virus-infected groups. The top 20 significantly enriched KEGG pathways were shown in the bubble diagram. The difference in bubble size means that the number of target genes enriched in the pathway is different and, the larger the bubble, the more target genes are enriched. The bubble color represents the *P* value, and the color change from red-green-blue-purple represents a small to large transition of the *P* value. The smaller the *P* value, the more significant the difference. The expression of putative target genes involved in this KEGG pathway is inversely related to the expression of their targeting miRNAs.

**FIGURE 7:**
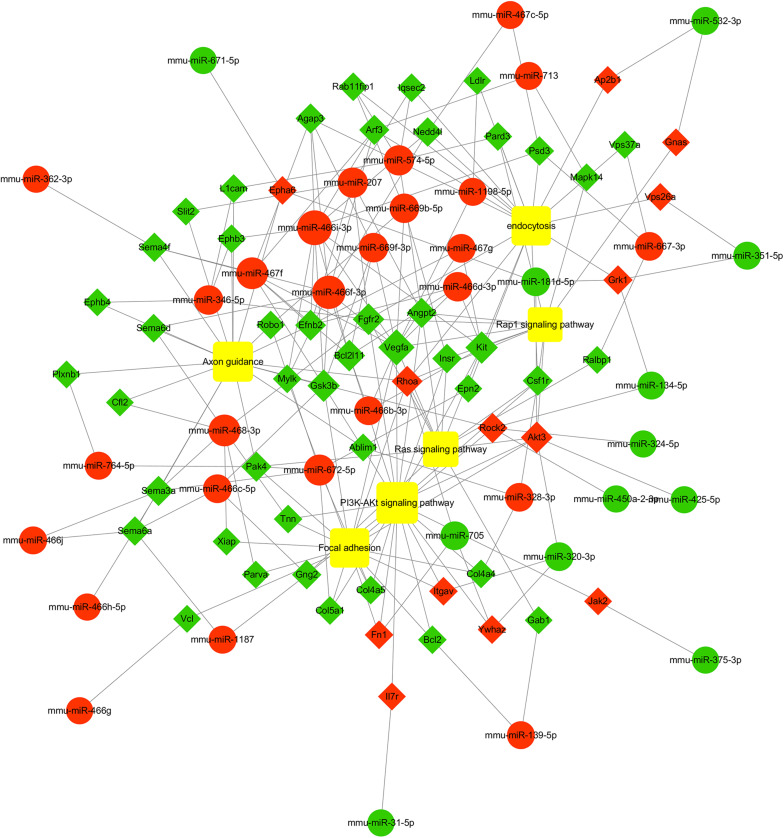
miRNA-mRNA interaction network of intersectional inversed genes of miRNAs common to the three PB2-variant groups. Circles indicate identified miRNAs, while diamond represents the corresponding target genes. Red indicates up-regulated miRNAs or mRNAs while green indicates down-regulated miRNAs or mRNAs. The relationship between miRNAs and genes is shown connected by gray lines. The larger the circle is, the greater the degree to which the miRNA or gene involved in the related pathway. Six significant enriched pathways associated with influenza virus infection were depicted using the yellow squares. More details about miRNA-mRNA involved in these six pathways are shown in Supplementary Table S11.

To further study the role of the identified differentially expressed miRNAs, we first predicted the miRNA target genes via MicroRNA.org, Targetscan, and PITA databases. A total of 9213 target mRNAs were predicted from the intersection of the three databases. The target mRNAs that were both predicted by the bioinformatics programs and were also target mRNAs confirmed by transcriptome sequencing were used as the initial putative target genes. Then, the initial putative target genes that did not demonstrate a negative regulatory relationship between miRNA and mRNA-target were excluded. Finally, the differentially expressed target mRNAs that passed through these screens were subject to signaling pathway analysis using Gene Ontology (GO) and Kyoto Encyclopedia of Genes and Genomes (KEGG). GO analysis results showed that the five most-enriched terms in the biological processes category were nervous system development, axon guidance, cell-cell adhesion, protein phosphorylation, and transcription. The top five enriched terms in the cellular component were cytoplasm, nucleus, cell-cell adhere junction, focal adhesion, and nucleoplasm. In the molecular function category, the top five terms were protein binding, cadherin binding involved in cell-cell adhesion, chromatin binding, kinase activity, and protein kinase binding. The top 10 GO terms were described in Figure 5 (More details can be seen in Supplementary Tables S6–S8). This data could provide evidence that these miRNAs potentially regulate genes involved in numerous processes during infection including gene transcription, expression, and activation. KEGG analysis of these target mRNAs showed that the putative target genes of the selected common miRNAs were enriched into 183 KEGG signaling pathways, and the top 20 signal pathways with the most enriched genes were depicted as bubbles (Figure 6). (More details about KEGG terms can be seen in Supplementary Table S9). Among those 20 signaling pathways, all were involved in the modulation of influenza virus infection signal pathways including PI3K-AKt signal pathway, Rap 1 signal pathway, Ras signal pathway, endocytosis, tight junctions, and axon-guiding signaling pathway. These six pathways were selected and the miRNA-mRNA regulatory network was generated based on the previously selected miRNA-mRNA relationship pairs. The miRNA-mRNA interaction diagram was shown in Figure 7. There were 38 differentially expressed miRNAs and 58 differentially expressed mRNAs involved in these six pathways. The pathways implicated in influenza virus infection were enriched in all of the groups. Collectively, these identified miRNAs could regulate the genes involved in the modulation of influenza virus pathogenesis.

### Functional Enrichment Analysis of Specific Differentially Expressed miRNAs

**FIGURE 8:**
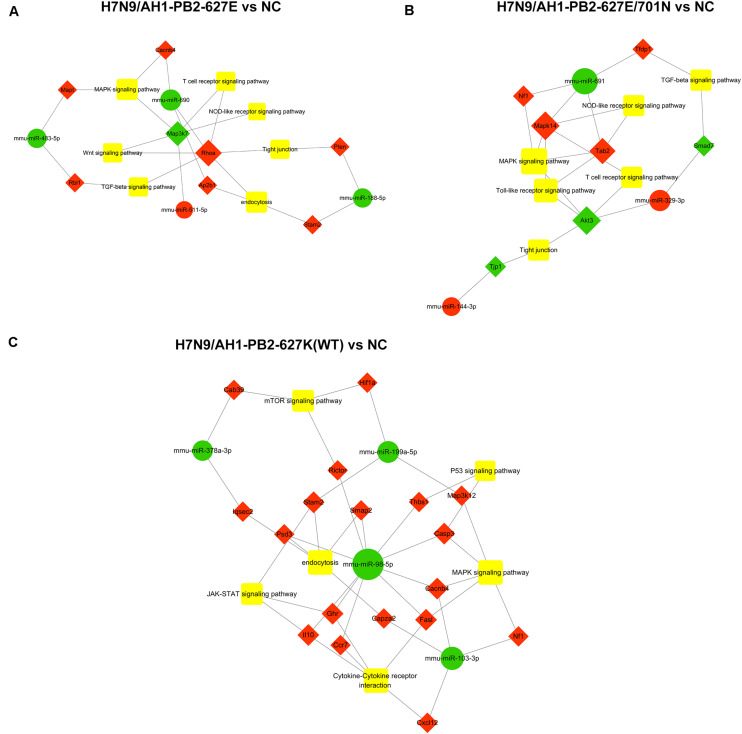
Global analysis of specific differential expressed miRNAs in mouse lung infected with H7N9 and its mutant influenza viruses compared with negative control group (NC). miRNA-mRNA interaction network in three different infected groups was built according to the specific miRNAs and their oppositely expressed genes. Green circle indicates miRNAs while red diamond represent the corresponding target genes. The relationship between miRNAs and genes is connected by gray lines. The larger the circle is, the greater the degree to which the miRNA or gene involved in the related pathway. **(A)** in H7N9/AH1-PB2-627E infected vs. NC group; **(B)** in H7N9/AH1-PB2-627E/701N infected vs. NC group; **(C)** in H7N9/AH1-PB2-627K(WT) infected vs. NC group.

The specific differentially expressed miRNAs induced by H7N9/AH1-PB2-627K(WT) and its mutant viruses H7N9/AH1-PB2-627E and H7N9/AH1-PB2-627E/701N infection groups were shown in Tables 2, 3. The Venn diagram in Figure 3B showed that for H7N9/AH1-PB2-627K(WT) a total of 12 specific miRNAs were significantly expressed in infected mice lung at day 3 post-infection. The groups infected with H7N9/AH1-PB2-627E and H7N9/AH1-PB2-627E/701N were identified carrying 33 and 12 specific differentially expressed miRNAs, respectively. The predicted target mRNAs of the confirmed specific miRNAs in each group, as well as the differentially expressed mRNAs in each group identified by transcriptome data, were selected for KEGG signaling pathway enrichment analysis. The signaling pathways directly related to influenza virus infection that were identified in each group were depicted in Supplementary Table S10, and the miRNA-mRNA regulatory network map was subsequently drawn in Figure 8. Bioinformatics analysis demonstrated that some specific miRNAs were prominent in regulating the replication and pathogenesis of each group of mutant viruses in mammals. mmu-miR-188-5p, mmu-miR-511-5p, mmu-miR-483-5p, and mmu-miR-690 were found only during H7N9/AH1-PB2-627E replication in mice. The specific host miRNAs involved in modulating H7N9/AH1-PB2-627E/701N virus replication were mmu-miR-691, mmu-miR-329-3p, and mmu-miR-144-3p. Lastly, the specific host miRNAs that play important regulatory roles in the H7N9/AH1-PB2-627K(WT) virus-infected group were mmu-miR-98-5p, mmu-miR-103-3p, mmu-miR-199a-5p, and mmu-miR-378a-3p. Therefore, the data could provide evidence that there is a close relationship between miRNA, host gene expression and viral pathogenesis.

### qRT-PCR to Verify the Relative Expression of Differentially Expressed miRNAs

**FIGURE 9:**
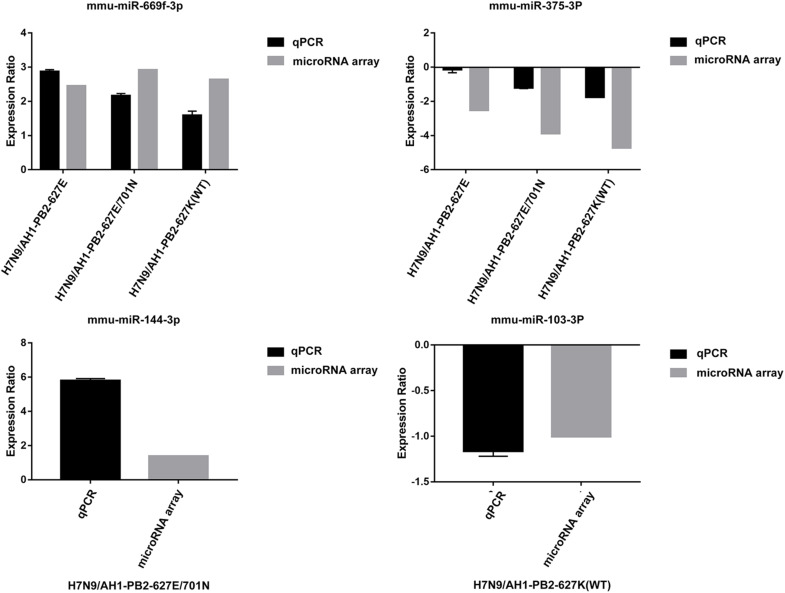
Comparison between microarray data and qRT-PCR results.

In order to verify the accuracy of miRNA chip sequencing results, two common differentially expressed miRNAs and two specific differentially expressed miRNAs were randomly selected for reverse transcription and qPCR verification, the results were shown in Figure 9. The expression trend of the selected miRNAs were consistent with the sequencing results. mmu-miR-669f-3p and mmu-miR-375-3p showed up-regulation and down-regulation, respectively, after mice were infected with the three viruses. The expression of the specific mmu-miR-144-3p was up-regulated following H7N9/AH1-PB2-627E/701N virus infection, while the specific mmu-miR-103-3p was down-regulated following the H7N9/AH1-PB2-627K(WT) virus infection. These results verify the accuracy of the sequencing results.

## Discussion

A substantial number of studies have shown that the role of miRNAs have been indicated as crucial in fine-turning avian influenza virus (AIV) infection process (Nguyen et al., 2018). Accumulating evidence suggests that H7N9 virus infection can alter host miRNA expression, however, little is known regarding miRNA and mRNA involved in infections by IAVs with key amino acid changes in PB2, and few integrated analyses of differentially expressed miRNAs and mRNAs following avian and mammalian influenza virus infections have been performed. In the current research, the pathogenicity of avian-type H7N9/AH1-PB2-627E, human-adapted H7N9/AH1-PB2-627K(WT), and mammalian-adapted viruses H7N9/AH1-PB2-627E/701N was measured by observing changes in mouse body weight, mortality, infected mouse lung viral titer and histopathology on the 3rd and 5th day post-infection. These observations provided proof that the pathogenicity of avian influenza virus H7N9/AH1-PB2-627E was significantly milder than that of H7N9/AH1-PB2-627K(WT) and H7N9/AH1-PB2-627E/701N. In addition, miRNA microarray coupled with transcriptome sequencing technology were used to screen the differentially expressed miRNAs and mRNAs caused by the infection in lung tissues of mice with these three viruses. In this study, we have systematically identified and compared, for the first time, the common and specific differentially expressed miRNA and mRNA expression profiles of mice lung tissues infected with PB2-variant viruses. This work lays the foundation for understanding the role of those host miRNAs that fluctuate in response to influenza virus infection in general, as well as those host miRNAs that react specifically when an interspecies influenza infection occurs.

Previous studies have confirmed influenza virus infection could induce differentially expressed miRNAs and mRNAs (Li et al., 2011). In this study, 220 common differentially expressed miRNAs and 2142 common differentially expressed mRNAs were significantly changed in mice infected with three different mutant-PB2 viruses. There were 34, 13, and 12 strain-specific differentially expressed miRNAs and 1503, 3712, and 3144 specific differentially expressed mRNAs that significantly changed in mice infected with avian-like virus H7N9/AH1-PB2-627E, human-like virus H7N9/AH1-PB2-627K(WT), and mammalian adapted virus H7N9/AH1-PB2-627E/701N, respectively. Several reports showed that host miRNAs were differentially expressed after infection with influenza virus, and miRNAs have been reported to regulate the antiviral immune responses to influenza virus (Nguyen et al., 2018). For example, the expression of miR-375-3p in the spleen of mice infected with A/Swine/Guangdong/2/2012 (H1N1) virus was significantly down-regulated (Huang et al., 2015). In our study, we also found the same expression trend of this miRNA, which suggested that miR-375-3p might be a universal miRNA down-regulated during influenza virus infection. Another study revealed that the over-expression of miR-199a could inhibit hepatitis C virus (HCV) genome replication (Murakami et al., 2009). Our results were consistent: miR-199a was clearly down-regulated in all three infected groups. miR-199a can also regulate cell proliferation and survival (Jiugang et al., 2014), and furthermore, it serves as a negative regulator of the TGF-β signaling pathway (Zhang et al., 2012). From the above, we conclude that miR-199a may be vital in the influenza virus infection process. The results of previous literature revealed that the ectopic expression of miR-144 could increase the replication of influenza virus in primary mice lung epithelial cells by targeting TRAF6 mRNA (Rosenberger et al., 2017). In this work, the expression profile and qPCR results proved that miR-144 was upregulated dramatically in the H7N9/AH1-PB2-627E/701N virus infected group, supporting the results of previous studies. And overexpression of mmu-miR-144-3p could substantially decreased the luciferase activities of the cullin 5 (Clu5) and early endosome antigen 1(Eea1) 3′UTR (Supplementary Figure S4). We suspected that this miRNA can also modulate H7N9/AH1-PB2-627E/701N virus replication through another eventful pathway. Literature demonstrates that miR-483 becomes dramatically up-regulated in mouse lung tissues infected with of H1N1, H5N1 or H7N9; it also shows that the over-expression of miR-483 could decrease viral replication by targeting the transcript of RING-finger protein 5. In our study, however, miR-483 was significantly down-regulated in the H7N9/AH1-PB2-627E infected group, an expression trend contrary to previous research results. We hypothesized that the reason for this results is that one virus, H7N9 (Anhui), has mammalian characteristics and that the other virus, H7N9/AH1-PB2-627E, has avian characteristics, with a glutamic acid at position 627 of the PB2 gene. It was reported that miR-188-3p was involved in the activation of NF-κB pathway (Meng et al., 2019) and mediates the PI3K/AKT pathway (Xue et al., 2018), both of which are significant immune-related pathways and also related to apoptosis (Wang et al., 2018). This work also identified the same miRNA although different target genes were predicted; we suspected that this miRNA might be important for resisting viral invasion via broad mechanisms. Other research identified miR-511 as a potent modulator of human immune response (Tserel et al., 2011), and this work also identified as well, specifically in the H7N9/AH1-PB2-627E infection group. The *Akt3* gene was identified as a differentially expressed gene in the specific miRNA-mRNA regulatory network of mice lung tissues infected with H7N9/AH1-PB2-627E/D701N virus. Akt3 is involved in the activation and inhibition of many signaling pathways, such as MAPK and Toll-like receptors, T cell receptors, and tight junction signaling pathways. It has been reported that the *Akt* gene has three types, namely *Akt1*, *Akt2*, and *Akt3*, and that their sequences and structures share high homology and even the same activation mechanism (Shi et al., 2016). Previous studies have shown that *Akt1* and *Akt2* are distributed in various parts of the body and can be activated by HCV infection (Street et al., 2004; Jackel-Cram et al., 2010). *Akt3* is capable of inhibiting the proliferation and migration of hepatocellular carcinoma, and its mRNA is a known target of miR-582-5p (Zhang et al., 2015) and miR-424 (Yang et al., 2015). It can also affect the viral cycle of the HCV virus and its viral transcription process. Our data strongly supported the results of previous studies, indicating these differentially expressed miRNAs play an important role in the infection of influenza virus or in the cross-species transmission of avian-origin influenza virus to a mammalian host. Therefore, further study the functions of these differentially expressed miRNAs and mRNAs is a future direction of research. Additional work is needed to clarify the mechanisms behind how these common miRNAs mediate host-virus interactions during influenza virus infection and how those specific miRNAs regulate the cross-species transmission of influenza virus.

The different miRNA and mRNA expression profiles identified in the three different influenza virus infected groups indicate that the pathogenicity of these viruses were different. The three PB2-variations alone could induce host cells to produce common differentially expressed miRNAs and specific differentially expressed miRNAs. Previous studies showed that common and specific differentially miRNAs and mRNAs were observed in A549 cells infected with human-origin H3N2 seasonal influenza A virus, swine-origin H1N1 influenza A virus or avian-origin H3N2 influenza a virus, which may be highly correlated with the molecular mechanisms of influenza virus infection. We hypothesized that these common differentially expressed miRNAs play a crucial role in the replication of influenza virus, and that specific differentially expressed miRNAs were indispensable for the regulation of cross-species transmission mechanisms of influenza viruses.

In this study, the integrated analysis of miRNA and mRNA expression showed that one miRNA can target several mRNAs, and one mRNA could be regulated by several miRNAs. For example, miR-466i-3p down-regulated the expression of *Agap3*, *Bcl2l11*, *Vegfa*, *Arf3*, *Gsk3b*, *Psd3*, *Mylk*, *Kit*, *Nedd4l*, *Efnb2*, and *Ephb3*, while *Akt3* could be up-regulated by miR-320-3p, miR-181d-5p, and miR-425-5p, indicating that these miRNA-mRNA networks were complicated and ordered. Previous studies showed that TGF-β signaling pathway (Schultz-Cherry and Hinshaw, 1996; Jones et al., 2005) and JAK-STAT signaling pathway (Kumar et al., 2018) participate in the processes of cell cycle control, differentiation, apoptosis, and innate immunity. Our pathway enrichment analysis results also identified the same pathways, indicating that some of the differentially expressed miRNAs induced by the three PB2-variant viruses may affect the pathophysiological process of influenza virus infection.

## Conclusion

In summary, this study performed the comparative integration analysis of miRNA-mRNA induced by three different H7N9 influenza viruses with one key amino acid change in a mouse model for the first time. From the infected groups of all three viruses, 38 and 58 common miRNAs and mRNAs were identified which were essential for viral replication or for restricting viral invasion. Within each group, some specific miRNAs were also identified: miR-188,511,483, and 690 were specific to H7N9/AH1-PB2-627E infection; miR-691,329, and 144 for H7N9/AH1-PB2-627E/701N infection; and miR-103,199a, and 378a for H7N9/AH1-PB2-627K(WT) infection, all of which are potentially of importance to influenza virus pathogenesis. This work provides a new perspective for further exploration of the regulatory mechanisms of these miRNAs and thus to the development of novel anti-influenza drugs.

## Data Availability Statement

The raw data of miRNA chip can be found here: https://www.ncbi.nlm.nih.gov/geo/subs/ and the GEO accession number: GSE150782. The original transcriptome data can be seen: https://submit.ncbi.nlm.nih.gov/subs/ with accession number C2:SRR12131309; C3: SRR12131308; C4:SRR12131305; E3:SRR12131304; E4:SRR12131303; E8:SRR12131302; EN1:SRR12131301; EN2:SRR12131300; EN4:SRR12131299; WT1:SRR12131298; WT2:SRR12131307; and WT3:SRR12131306. The C2, C3, and C4 represent three biological repeats of NC group in our manuscript; E3, E4, and E8 are three replicates of H7N9/AH1-PB2-627E infection group; EN1, EN2, and EN4 are three samples from H7N9/AH1-PB2-627E/701N infection group; WT1, WT2, and WT3 represent three repeated samples in H7N9/AH1-PB2-627K(WT) infected group.

## Ethics Statement

The animal study was reviewed and approved by the Nanjing Agricultural University Experimental Animal Welfare Ethics Committee with the approval ID is SYXK2017-0007.

## Author Contributions

YG and JP designed the experiments. YG, NH, MT, MF, QL, ZL, and TS carried out the experiments. YG, NH, JH, HX and YZ analyzed the result data. YG wrote the manuscript. All authors read and approved the final manuscript.

## Conflict of Interest

The authors declare that the research was conducted in the absence of any commercial or financial relationships that could be construed as a potential conflict of interest.
