# Supplementary material for: Integrated Analysis of microRNA-mRNA Expression in Mouse Lungs Infected With H7N9 Influenza Virus: A Direct Comparison of Host-Adapting PB2 Mutants
Source: Front Microbiol. 2020 Jul 28;11:1762. doi: 10.3389/fmicb.2020.01762 (PMC7399063; doi:10.3389/fmicb.2020.01762)
Supplement: Supplementary file 1 [file Data_Sheet_1.zip › Supplementary_Material.docx]

Supplementary Material

**Supplementary Figure S1.** Verification of randomly screened 12 differentially expressed miRNAs by qPCR method.

**Supplementary Figure S2.** The effect of miRNAs on WSN influenza virus replication.

**Supplementary Figure S3.** Verification of randomly screened 16 differentially expressed miRNAs by qPCR method.

**Supplementary Figure S4.** mmu-miR-144-3p targets the 3’UTRs of Clu5 and Eea1.

**Supplementary Table S1.** The primers applied in the qPCR method to verify the precision of selected miRNA.

**Supplementary Table S2.** All common up-regulated differentially expressed miRNAs identified in three PB2-variant viruses infected group compared to NC group.

**Supplementary Table S3.** All common down-regulated differentially expressed miRNAs founded in three PB2-variant viruses infected group compared to NC group.

**Supplementary Table S4.** All intersected up-regulated differentially expressed mRNAs founded in three PB2-variant viruses vs NC group.

**Supplementary Table S5.** All intersected down-regulated differentially expressed mRNAs identified in three PB2-variant viruses vs NC group.

**Supplementary Table S6.** Biological process(BP) enrichiment analysis results of selected differentially expressed target genes of 220 common miRNA was conducted.

**Supplementary Table S7.** Cellular component(CC) enrichiment analysis results of selected differentially expressed target genes of 220 common miRNA was detailed.

**Supplementary Table S8.** Molecular function(MF) enrichiment analysis results of selected differentially expressed target genes of 220 common miRNA was depicted.

**Supplementary Table S9.** All KEGG item of putative differentially expressed target genes of 220 common miRNA was described.

**Supplementary Table S10.** The selected negative miRNA-mRNA pairs in each infected groups.

**Supplementary Table S11.** The detailed information of miRNA-mRNA network in the selected signaling pathway which was related to influenza virus infection.
