# Supplementary figures and images for: Integrated Analysis of microRNA-mRNA Expression in Mouse Lungs Infected With H7N9 Influenza Virus: A Direct Comparison of Host-Adapting PB2 Mutants
Source: Front Microbiol. 2020 Jul 28;11:1762. doi: 10.3389/fmicb.2020.01762 (PMC7399063; doi:10.3389/fmicb.2020.01762)

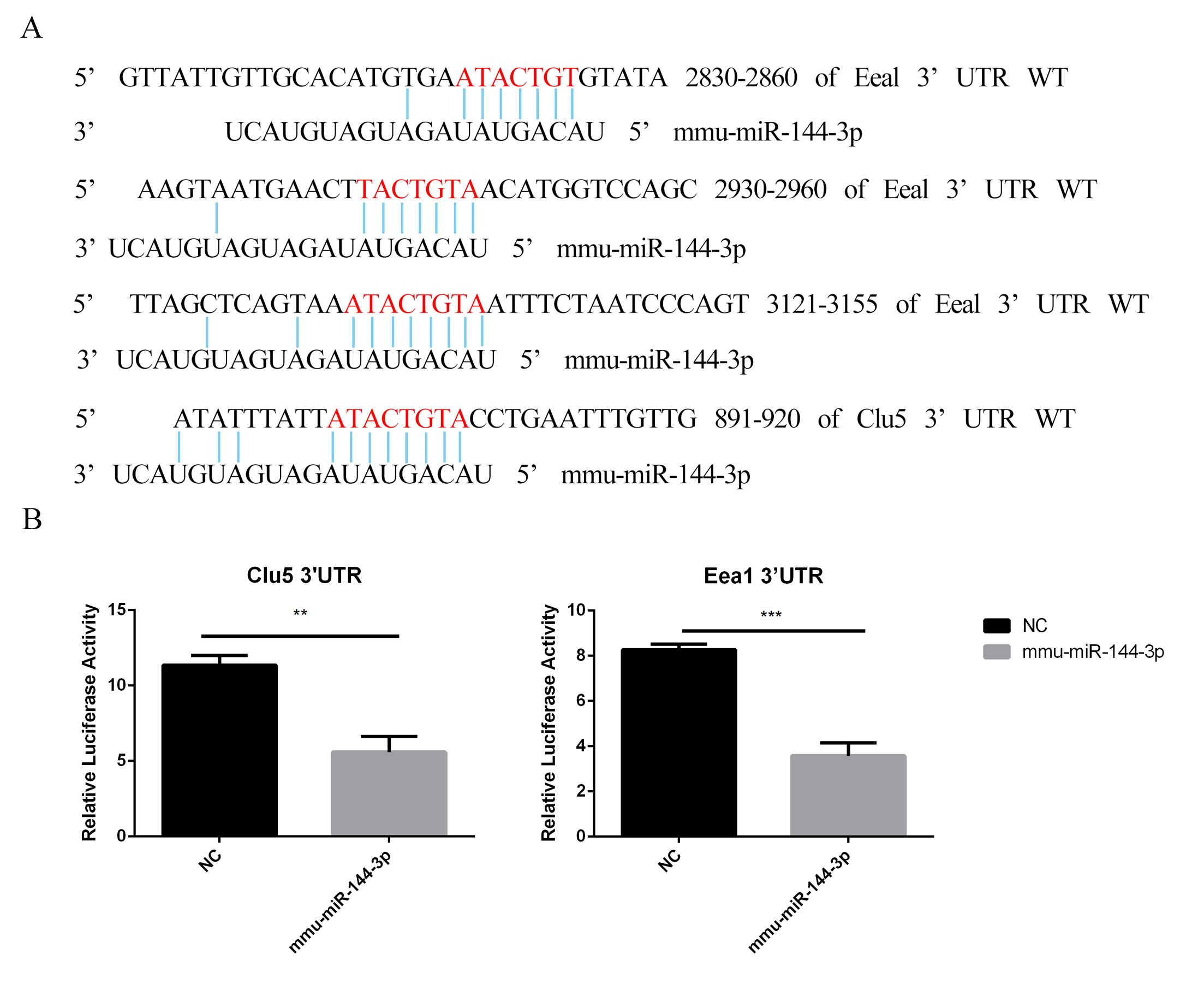

Supplement: Supplementary file 1 [file Data_Sheet_1.zip › Fig S4 .tif]
